# Progress of Molecular Display Technology Using *Saccharomyces cerevisiae* to Achieve Sustainable Development Goals

**DOI:** 10.3390/microorganisms11010125

**Published:** 2023-01-03

**Authors:** Seiji Shibasaki, Mitsuyoshi Ueda

**Affiliations:** 1 Laboratory of Natural Science, Faculty of Economics, Toyo University, Hakusan, Bunkyo-ku, Tokyo 112-8606, Japan; 2 Graduate School of Agriculture, Kyoto University, Kitashirakawa-Oiwakecho, Sakyo-ku, Kyoto 606-8502, Japan

**Keywords:** yeast, genetic engineering, molecular display technology, sustainable development, sustainable development goals (SDGs)

## Abstract

In the long history of microorganism use, yeasts have been developed as hosts for producing biologically active compounds or for conventional fermentation. Since the introduction of genetic engineering, recombinant proteins have been designed and produced using yeast or bacterial cells. Yeasts have the unique property of expressing genes derived from both prokaryotes and eukaryotes. *Saccharomyces cerevisiae* is one of the well-studied yeasts in genetic engineering. Recently, molecular display technology, which involves a protein-producing system on the yeast cell surface, has been established. Using this technology, designed proteins can be displayed on the cell surface, and novel abilities are endowed to the host yeast strain. This review summarizes various molecular yeast display technologies and their principles and applications. Moreover, *S. cerevisiae* laboratory strains generated using molecular display technology for sustainable development are described. Each application of a molecular displayed yeast cell is also associated with the corresponding Sustainable Development Goals of the United Nations.

## 1. Introduction

In biochemical studies, yeasts are considered to be a representative model of eukaryotic microbes. The budding yeast *Saccharomyces cerevisiae* contains only 6611 genes (https://www.yeastgenome.org, accessed on 16 December 2022); however, it has been used for investigating gene and protein functions in recent molecular biology studies [1,2,3,4,5].

Moreover, the use of *S. cerevisiae* as catalysts in various fermentation industries has a longer history than their application in modern bioscience and biotechnology. For instance, yeasts have been employed in the production processes of fermented foods, such as Japanese sake [6,7], beer [8], bread [9,10], and miso [11,12]. Thus, *S. cerevisiae* has been related to our diet for a long time and gained a generally regarded as safe (GRAS) status [13,14], although a few strains are pathogenic [15]. Since the advent of genetic engineering, *S. cerevisiae* has been used for producing valuable compounds. Several recombinant proteins have also been developed as pharmaceuticals using yeast cells [16,17]. Given their usefulness and safety, *S. cerevisiae* can help us achieve sustainable development.

In this century, biotechnology is expected more than ever to solve socioeconomic issues. Although industrialization has made human life convenient, it has also given rise to environmental issues, such as global climate change, food shortage, and pollution. To overcome these problems, the United Nations proposed 17 Sustainable Development Goals (SDGs) for maintaining human health and establishing a sustainable society [18]. Natural resources or products form the basis of and are consumed during the economic activities in developing and developed countries, causing industrial and environmental problems that need to be mitigated. Considering that biotechnology has improved the quality and scale of industrial and agricultural production, it may also provide several opportunities for sustainable development. The latest biotechnology development in manufacturing not only improves production efficiency, but also promotes international trade and cooperation toward mutual development [19]. Among various biotechnologies, microbial biotechnology is expected to achieve SDGs [20]. Microbiology and microbial technology are considered the key to achieving SDGs via the eradication of infectious diseases, provision of clean water, food security, maintenance of terrestrial and marine biodiversity, and utilization of biofuels [21]. To attain such goals using biotechnology, convenient and economic methods should be implemented worldwide. Herein, we describe the molecular display system, which is a recently developed biotechnique that can help achieve such convenience and economic efficiency.

## 2. General Description of Molecular Display Technology

Molecular display technology or cell-surface engineering is a biotechnological method of genetic engineering that is focused on the cell surface. The phage display system proposed by Smith has the longest history among molecular display systems [22]. It was employed in searching for a clone that can bind to a target compound or investigating protein interactions and is still widely used today [23,24]. A phagemid vector encoding a foreign protein to be displayed as a fusion of the coat protein can be introduced into a phage, resulting in a library consisting of 10^12^ clones. Next, the panning process is conducted to select positive clones that can bind to target molecules from this phage library; this process includes immobilization of the target molecule on a solid phase, incubation of a library and the target molecule, removal of bound phages from solid phages, infection of *Escherichia coli* with the recovered phage, and amplification of the clones. This sequential panning cycle should be performed several times. In addition, bacterial cells have been developed and were shown to improve the phage display method, which uses complicated panning processes to isolate a clone capable of binding to a target. For example, surface display systems of foreign proteins using *Lactococcus lactis* [25], *Staphylococcus aureus* [26], and *E. coli* [27] as the host cell have been developed. In addition, unlike phage display systems, bacterial display systems were applied not only in the selection of protein clones that can bind to a target compound, but also in the establishment of whole-cell biocatalysts coupled with metabolic reaction in host cells [28].

Yeasts have been employed in molecular display technology for 20 years [29,30,31]. The yeast *S. cerevisiae* is useful as a host microorganism in genetic manipulation because it can produce and glycosylate foreign proteins derived from other eukaryotes. This yeast species also has the advantage of high-density cultivation in various media at a low cost. In addition, this yeast not only displays proteins derived from other eukaryotes but also different proteins on its cell surface, i.e., “co-display” [32]. As another characteristic of host cells, several auxotrophic markers can be used in the genetic manipulation of yeast cells for producing different recombinant proteins. Furthermore, flow cytometry or high-throughput screening is applicable for selecting target protein-displaying yeast cells [33,34,35]. Thus, molecular display or cell-surface engineering using the yeast cell surface has many important benefits and practical applications. Yeasts capable of displaying foreign proteins, especially *S. cerevisiae*, are called “arming yeasts” [36,37,38,39,40]. The principle of molecular display using yeasts and its applications to achieve sustainable development are describe in the subsequent sections.

## 3. Principle of Molecular Display Technology Using Yeasts

Regardless of the type of host cells selected for molecular display, the anchoring protein must be valid and effective. For example, OmpA has been investigated and used as an anchoring protein to display a foreign protein on the cell surface of *E. coli* [41,42]. It is usually used as a lipoprotein, which is fused to residues 46–159 of the OmpA porin protein family to anchor to the *E. coli* cell wall envelope. Moreover, the cA domain of AcmA (a major autolysin from *L. lactis*) [25] and PgsA from *Bacillus subtilis* [43] have shown efficacy in displaying several foreign proteins on the cell surface of *Lactobacillus* sp.

**Figure 1:**
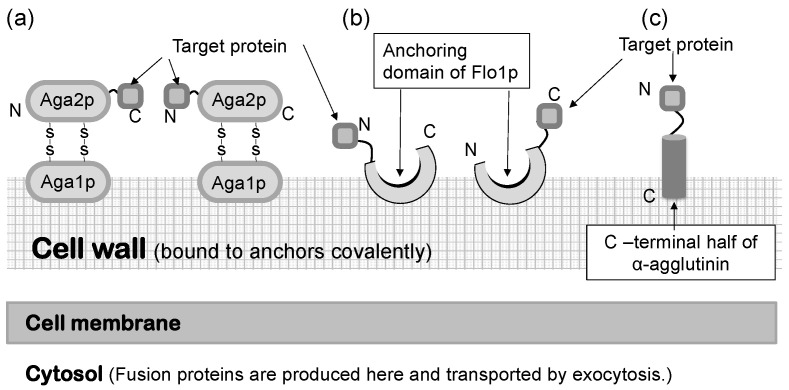
Molecular display systems in *Saccharomyces cerevisiae*. A target protein is immobilized by several types of anchoring proteins. (**a**) a-agglutinin-based display system. (**b**) Flo1p-based display system. (**c**) α-Agglutinin-based display system. Figures were adapted from [47].

In a *S. cerevisiae* molecular display system, several anchoring proteins can be used to immobilize the target protein (Figure 1). The Flo1 protein can anchor the target protein in two ways. It is produced and attached on the yeast cell surface for flocculation. In molecular display technology, the target protein to be displayed on the yeast cell surface is usually fused at its N-terminal to the C-terminal of the Flo1 protein. Moreover, Aga1-Aga2 proteins, which are also used for displaying the target protein, are favorable because they can display a C-terminal-free protein on the yeast cell surface. If the active site of the target protein is located in the N-terminal, or if its C-terminal conformation does not affect the function of the target protein, α-agglutinin can be used.

*Saccharomyces cerevisiae* has a thick, hard cell wall that consists of β-linked glucans and mannoproteins. The cell wall is located outside the plasma membrane and consists of an internal skeletal layer of glucan composed of β-1,3- and β-1,6-linked glucose and a fibrillar or brush-like outer layer composed predominantly of mannoproteins [44]. There are two types of mannoproteins in the thick cell wall [45]. One is loosely bound to the cell wall with non-covalent bonds and can be extracted using sodium dodecylsulfate (SDS). The other mannoprotein can be extracted by using β-1,3- or β-1,6-glucanase. Cell wall proteins illustrated in Figure 1 are glucanase-extractable mannoproteins covalently linked with the glucan layer of the cell wall.

**Figure 2:**
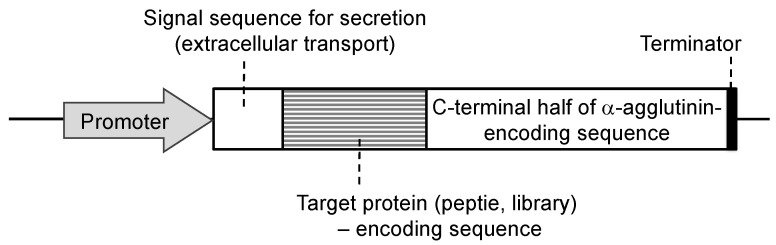
Fusion gene construction for cell-surface display of metal-binding protein and peptide on yeast cell surface. Secretion signal sequence is necessary for extracellular transport of a fusion protein.

Since the development of yeast molecular display systems, α-agglutinin has been widely investigated and used as an anchoring protein [46,47,48]. Mating-type alpha cells have α-agglutinin protein on their cell surface, which functions during mating with other types of *S. cerevisiae* cells. This protein is covalently bound to the cell wall, causing the target protein to be fused and stably displayed on the cell surface. Although α-agglutinin itself has a predicted length of 650 amino acids before processing, genetic engineering allows the fusion of the target protein to the C-terminal half (320 amino acids) of α-agglutinin as a cell wall anchor. In the genetic preparation for molecular display, a gene encoding foreign protein is placed between a gene encoding secretion signal sequence and a gene encoding the C-terminal half of α-agglutinin (Figure 2). This genetic construct is expressed under a suitable promoter sequence in a plasmid when it is successfully incorporated by the host yeast cells. In the following sections, several applications of molecular display systems of *S. cerevisiae* with α-agglutinin are described.

## 4. Applications in Environmental Processes

To achieve a sustainable society, solutions for various environmental issues must be created by science and technology to assist the self-repair function of the earth. In SDGs, several indicators are related to the improvement of environmental issues. Indeed, agrotechnology or biotechnology is considered to be able to achieve four SDGs: namely, clean water and sanitation (SGD6), affordable and clean energy (SDG7), responsible consumption and production (SDG12), and climate action (SDG13) [49].

Industrial activities and developments involve the release of chemicals and heavy metals from mines into the environment. For example, in the 1880s, pollutants from the Ashio copper mine in Japan devastated the surrounding environment, and the pollution remained even after the mining activities had stopped [50]. Similar problems have occurred in other countries. For example, in a river in Brazil, it was estimated that several tons of copper have been released by waste rocks into the water over the last 30 years [51]. The toxicity of copper has been shown in reproductive performance. In addition to copper, other heavy metals such as cadmium, mercury, arsenic, and zinc also affect several reproductive functions in both males and females [52]. Moreover, these heavy metals are not only found in mines but also in waste electronic devices [53].

Bioremediation technology is expected to repair contaminated environments [54]. Bioremediation is aimed at the removal or recovery of hazardous substances from biological materials, including living or dead microorganisms and their components, seaweeds, plant materials, and agricultural wastes [55]. For instance, plants have the ability to neutralize contaminants in soil through absorption and accumulation in their roots [56,57]. Considering the convenience of their preparation as adsorbents, microorganisms have several advantages over plant roots since microbial cells can proliferate faster than plant cells. A microbial cell that acquires a high ability to bind heavy metal ions would be a good bioadsorbent for removing heavy metal ions in water environments.

**Figure 3:**
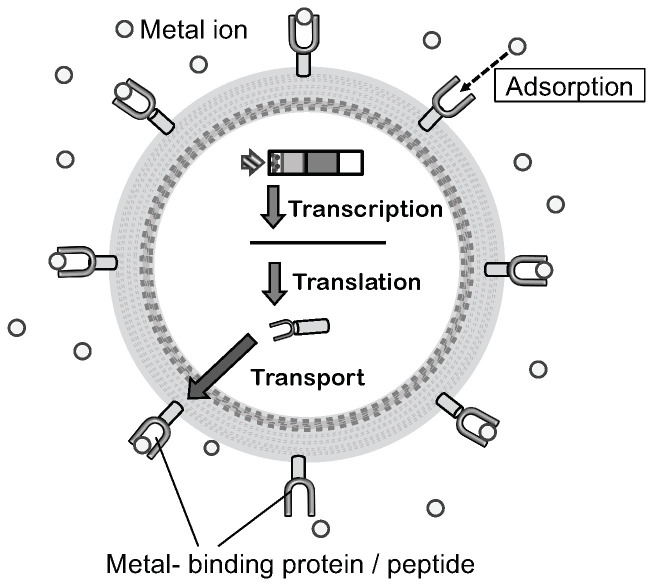
Mechanism of molecular display and constructional model bioadsorbent. Displayed metal-binding protein or peptide on the cell surface can bind a metal ion. The figure was adapted from [46].

Hexa-histidine peptide (His)_6_ is a well-studied moiety that can bind metal ions. Nickel chelate column is also widely and commercially available when compared to this peptide-tagged protein. Similarly, yeast metallothionein protein is known to possess metal-binding ability. To prepare a bioadsorbent using a microbial cell, these peptides and a protein are displayed on yeast cells using molecular display technology (Figure 3) [58,59]. Metal-binding molecule-displaying yeast cells are examined for their adsorption ability by incubation in a solution containing Ni^2+^, Cu^2+^, or Cd^2+^. These heavy metal ions recovered on the cell surface were desorbed and recovered by treatment with ethylenediaminetetraacetic acid (EDTA).

In addition to recovery techniques for heavy metal ions, recovery tools for rare metal ions are also required. It is known that several rare metals, such as vanadium, cobalt, selenium, manganese, and molybdenum, are important for maintaining the physiological function of diverse living organisms from microorganisms to humans [60,61]. Furthermore, rare metal has attracted attention in electric industries, and the importance of rare metal production is increasing each year. Urban mining is a recent idea based on the recovery of disposed electronic devices, and it reflects industrial importance [62]. Extraction of rare metals from urban mines is considered to be an environmentally friendly method when compared with natural mining, which uses fossil energy [63]. Currently, the extraction of rare metals from urban mines has been carried out using chemical reagents. A recovery process of rare metals that uses low amounts of chemical reagents would provide us with a less energy-consuming and more environmentally friendly solution in this field. To demonstrate that molecular display technology can be utilized in the development of bioadsorbents for rare metal ions, a molybdenum ion binding protein was displayed on the yeast cell surface. Molybdenum uptake is well understood in *E. coli* and other bacteria and is regulated by the transcription factor ModE, which binds to molybdate [64]. Therefore, ModE was displayed on the yeast cell surface and investigated for its binding ability to molybdate [65]. After confirmation of the successful display of this protein, the binding of molybdate to the ModE protein displayed on yeast was evaluated in an aqueous solution. The absorbed molybdate on the yeast cell surface was effectively released by treatment with papain. In addition, the specificity of the displayed ModE mutant protein (T163Y) for tungstate was enhanced by 9.3-fold when compared to that of the wild-type ModE protein displayed on the yeast cell surface [66].

Thus, by using molecular display technology and metal-binding peptides, yeast cells can be converted to metal-binding adsorbents. Furthermore, cellular tolerance to the heavy metal ions can be enhanced by the display of metal-binding molecules on yeast cell surfaces [67].

In addition to the pollution caused by heavy metal ions, that caused by other molecular contaminants in the environment must also be solved. It has been revealed that endocrine-disrupting compounds (EDCs) perturb the original function of the endocrine in mammals [68,69,70]. Increasing attention has been paid to EDCs concerning public health since they have the potential to disturb normal physiological pathways by interacting with steroid hormone receptors. Recently, the biodegradation potential of EDCs has been investigated [71]. For examining the abilities of chemical compounds to act as EDCs and bind to steroid hormone receptors, biochemical analytic tools have been created to evaluate the estrogenicity of EDCs [72]. Using a yeast molecular display system, a cell having the ligand-binding domain of the rat estrogen receptor (ERLBD) was produced as a novel assay tool of EDCs [73]. In the new assay system, fluorescently labeled 17β-estradiol (17-FE) was used as an analog of the natural ligand of ERLBD. Using this assay tool, the number of displayed ERLBDs was calculated, and it was very similar to that obtained for other proteins displayed on the yeast cell surface after the maximum display of the target protein using an α-agglutinin system. These findings suggest that ERLBD-displaying yeast would not only be useful for the assay of EDCs, but also for removing EDCs from the environment.

Yeast cells displaying a metal-binding protein or ERLBD can be conveniently and quickly prepared as bioadsorbents by cultivation. Therefore, they can be applied in the bioremediation of cleaning water in contaminated environments. By developing a reactor or a process suitable for these bioadsorbents, a novel technology can be created and contribute to SDG6: “sustainable management of water and sanitation for all”. In addition, rare metal-binding protein-displaying yeast could reduce the natural mine development and land degradation. Therefore, it could also contribute to SDG15: “halt and reverse land degradation and halt biodiversity loss”.

## 5. Applications as a Cell Sensor for Biosensing and Biomonitoring

**Figure 4:**
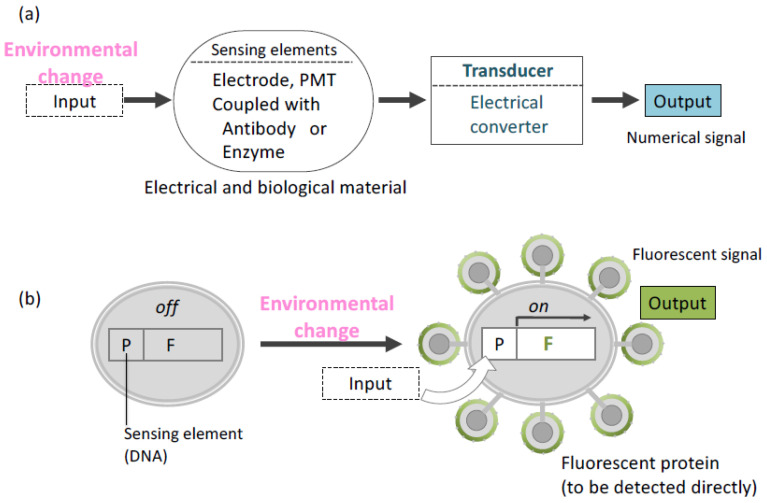
Overview of concepts about biosensing. (**a**) Conventional system (**b**) Cell sensor. In a cell sensor, “Environmental change” as input signal is converted to output signal at the identical place (cell). P: promoter, F: Gene encoding fluorescent protein.

Noninvasive monitoring is important for acquiring real-time information on the environment of living cells. The conventional biomonitoring or biosensing system consists of a sensing element and a transducing element (Figure 4) [74]. An enzymatic reaction and specific binding using an immunological molecule are representative of a sensing element. In addition, electronic devices have been used as a transducing element in biosensors [75]. Transducers are used to convert detected signals to numerical data. For a long time, these two elements were regarded as different parts in a biosensing system. Therefore, an invasive method is used to elucidate the biochemical condition and environment of the cell of interest.

Molecular display technology has unified sensing and transducing elements in one living cell [30]. A cell sensor created by this technology has been shown to capture and transduce information on the cell environment. In this sensing system, intra- or extracellular environment information is received by a promoter sequence, resulting in the expression of a protein to be displayed on the cell surface. Moreover, a fluorescent protein can be displayed by using a selected promoter. In the first trial of this cell sensor, glyceraldehyde-3-phosphate dehydrogenase (*GAPDH)* promoter [76] and jellyfish *Aequorea victoria* green fluorescent protein (GFP) [77] were selected as the sensing element and transducing element, respectively, in yeast cells [78]. The promoter was induced by glucose, allowing the fluorescence intensity detection from GFP on the yeast surface. Glucose concentration and fluorescence intensity were also correlated. In another trial, isocitrate lyase promoter (*UPR-ICL)* derived from *Candida tropicalis* [79] was introduced to detect glucose depletion using blue fluorescent protein (BFP). *UPR-ICL* usually represses the transcription of a gene in its sequence in the presence of glucose. The expression of BFP on the yeast cell surface was controlled by *UPR-ICL* [80]. Since GFP and BFP have different emission and excitation wavelengths [81], both proteins were displayed on an identical yeast cell surface, and the switching of those two promoters were affected by glucose concentration.

In addition to glucose, other nutrients can be detected by cell sensors created using molecular display technology. Ammonium and phosphate ions are important nutrients in growth media for yeast proliferation [82]. The *PHO5* promoter is the upstream sequence of the *PHO5* gene that encodes acid phosphatase. The transcription of *PHO5* is regulated by the extracellular concentration of inorganic phosphate. It is repressed in a high-phosphate medium and de-repressed in a low-phosphate medium [83]. The *MEP2* promoter is the upstream sequence of the *MEP2* gene that encodes ammonium ion transporter protein [84]. It is required for retaining NH^4+^ inside cells during growth in minimal nitrogen sources other than NH^4+^. In the presence of a good nitrogen source (glutamine, asparagine, or ammonium ion), the *MEP2* gene is repressed [85,86]. In this way, the *MEP2* and *PHO5* promoters are induced by ammonium and phosphate depletion, respectively. To detect the depletion of ammonium in the medium, the *MEP2* promoter was inserted into a cassette vector expressing enhanced yellow fluorescent protein (EYFP). The *PHO5* promoter was inserted into a promoter cassette vector expressing enhanced cyan fluorescent protein (ECFP) [87]. Next, flow cytometry analysis was used to detect ECFP or EYFP fluorescence on the yeast cell surface, which reflected the intra- and extracellular concentrations of phosphate or ammonium ions, respectively. The associations between the intra- and extracellular concentrations of each ion are evaluated from the changes in the fluorescence rate on the yeast cell surface.

The molecular display systems using variants of GFP described above were also effective in the monitoring of foreign protein production. To demonstrate the monitoring ability of yeast-displaying fluorescent proteins in a practical model of protein production, we take the *GAL1* promoter as an example. At the transcription level, the *GAL1* gene was induced by 1000-fold in the presence of galactose [88]. Molecular display of enhanced green fluorescent protein (EGFP) [89,90] on the yeast cell surface was well-controlled by the *GAL1* promoter, and two foreign proteins were produced intra- and extracellularly. Their production was monitored based on the fluorescence intensity of EGFP on the yeast cell surface [91]. An example of intracellular protein to be monitored is β-galactosidase, derived from *E. coli* [92]. Another example of a displayed foreign protein is human interferon omega (IFN-ω). The cytokine IFN-ω is secreted by virus-infected leukocytes as a major component of human leukocyte interferon [93]. It is also detected in the serum of patients with severe coronavirus disease 2019 (COVID-19) [94]. These two proteins were produced under the control of the same promoter, *GAL1*, in the EGFP-displaying yeast [91]. In the galactose-containing medium, the levels of β-galactosidase or IFN-ω produced were successfully monitored based on the fluorescence intensity of EGFP displayed on the yeast cell surface under the regulation of the *GAL1* promoter. In addition to the recombinant protein production, the intra- and extracellular concentrations of galactose, which was the sole carbon source, and the fluorescence intensity of EGFP displayed on the cell surface were monitored for 24 h, and their correlations were analyzed. The results suggested that this system can be used for noninvasive, fluorescence detection-based monitoring of protein production and medium components in bioprocesses. Since they use lower energy when compared to conventional sensing or monitoring systems, the abovementioned molecular display-based biomonitoring and biosensor systems can help us attain SDG9: “Build resilient infrastructure, promote inclusive and sustainable industrialization, and foster innovation,” especially in industries requiring the implementation of sustainable bioprocesses.

## 6. Application as a Tool in Biological Studies

**Figure 5:**
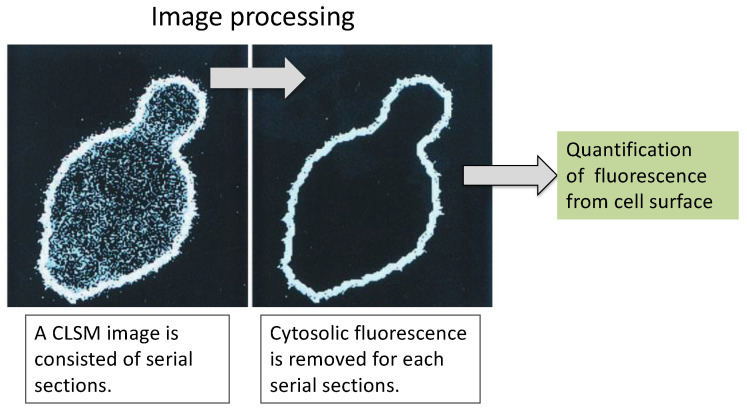
CLSM image processing for cell-surface fluorescence signal. (**Left**) Before processed serial section. (**Right**) Extracted surface fluorescence after processing. There is only a fluorescent signal for EGFP on the cell surface. The figure was adapted from [98].

The cell wall has long been a subject of biological research. In particular, advances in electron microscopy have enhanced our knowledge of the structure and biochemistry of the cell wall of *S. cerevisiae* [95,96]. Fluorescent proteins can also provide basic and important information on molecules displayed in an α-agglutinin system. EGFP, which has 35-fold higher fluorescence intensity than wild-type GFP [97], was displayed on the yeast cell surface. Since EGFP absorbs light of 488-nm wavelength emitted from an argon-ion laser, it is suitable for confocal laser scanning microscopy (CLSM) analysis. This facilitates precise evaluation of cell wall molecules. The number of EGFP molecules that can be displayed on the yeast cell surface was determined by CLSM analysis and image processing software (Figure 5) [98] to be approximately 1 × 10^4^ to 10^5^. This result corresponded well with the data obtained from fluorometry. This CLSM analysis coupled with image processing can be applied to various clinically obtained tissues or cellular samples for the quantification of surface proteins as a diagnostic maker and is expected to contribute to the achievement of SDG3: “healthy lives and promotion of well-being for all at all ages”.

## 7. Applications as a Whole-Cell Catalyst in Energy Production

The growing world population is expected to cause the depletion of energy resources unless we drastically change our lifestyle, such as stopping the use of fossil fuels. To achieve a sustainable society, biofuels are expected to replace fossil fuels [99,100]. In molecular display systems, various foreign enzymes are immobilized on the yeast cell surface. This system can produce a compound that is difficult to be converted by only yeasts. Moreover, the whole-cell catalyst produced by immobilizing enzymes on the yeast cell surface can be used repeatedly, more times than pure enzymes in industrial applications. The transformation of yeast cells into a whole-cell catalyst through the display of a recombinant enzyme on the cell surface can be adopted as a method for producing a renewable energy source, as described in this section.

Glucoamylase from *Rhizopus oryzae* was the first foreign enzyme displayed on the cell surface of an α-agglutinin system. This protein is an exo-type amylolytic enzyme that effectively cleaves α-1,4 and α-1,6-linkage of amylose from starch [101]. Glucoamylase-displaying yeast grew under aerobic conditions, using starch as a sole carbon source and producing ethanol [102]. An endo-type amylose from *Bacillus stearothermophilus,* which is known as α-amylase, was additionally displayed on the glucoamylase-displaying yeast [103]. This co-display of amylolytic enzymes led to the improvement of cell growth in starch-containing media. These studies were the first to demonstrate that molecular display technology provides effective methods for bioethanol production [104] and can contribute to achieving a carbon-neutral society owing to the use of disposed starch materials as a carbon source [105].

In addition to starch materials, cellulose is another possible material for bioethanol production. Recently, studies have focused on cellulosic materials as a raw material for bioethanol production since these materials do not interfere with world food availability [106]. Cellulose is a polysaccharide of the β-1,4-glycoside-linked linear chains of glucose. Cellulose contains crystalline and amorphous domains, and several kinds of hydrolytic enzymes are required for its degradation [107]. An endoglucanase (EG), a β-glucosidase, and a cellobiohydrolase are involved in cellulose degradation. BGL1 from *Aspergillus aculeatus* and endoglucanase II (EG II) from *Trichoderma reesei* were co-displayed on the yeast cell surface. This yeast was able to grow in a medium containing barley β-glucan as the sole carbon source. [108,109]. This process produced ethanol with lower energy than chemical processes and therefore would contribute to SDG7: “Ensure access to affordable reliable sustainable and modern energy for all”. In addition, these microbial processes can convert cellulosic materials derived from plants; thus, the enzyme-displaying yeast can facilitate effective carbon-neutral production of bioethanol and contribute to SDG13: “Take urgent action to combat climate change and its impacts”.

## 8. Applications for Production of Useful Nutrients

Isoflavones are naturally occurring flavonoids commonly found in *Glycine max* (soybean). They are garnering attention not only from scientists but also health-conscious individuals, owing to their physiological effects in preventing osteoporosis, hormone-related cancers, and coronary heart and neurological diseases [110].

In the fermentation process of soybean products, β-glucosidase derived from microorganisms, e.g., *Aspergillus brasiliensis* (*Aspergillus niger*)*, Aspergillus flavus var. oryzae* (*Aspergillus oryzae*)*,* and *Aspergillus pulverulentus*, are considered to be involved in the degradation of the saccharide form of isoflavone. In the production of Japanese miso, two types of β-glucosidase are known to participate in the enzymatic reaction that produces the aglycon form of isoflavone. Isoflavone aglycones are hydrolysates of isoflavone glycosides catalyzed by β-glucosidase and are highly effective in the prevention of several diseases mentioned above [110].

For the effective production of isoflavone, a yeast strain displaying β-glucosidase derived from *A. flavus var. oryza* was constructed. The ability of the strain to produce isoflavone aglycones from isoflavone glycosides was also examined [111]. Prior to genetic construction of the yeast display, putative genetic sequences of β-glucosidases were cloned from koji mold (*A. flavus var. oryzae*), considering that the soybean paste fermented by koji mold contains a high isoflavone content. BGL1-, BGL3-, and BGL5-encoding genes were isolated and displayed on the cell surface of sake yeast under the control of the *SED800* promoter. The strain displaying BGL1 showed the highest activity and had the broadest substrate specificity to isoflavone glycosides. These results suggested that the selection of the optimal isozyme led to the effective conversion of substrates into beneficial nutrients. Therefore, yeast molecular display may contribute to achieving SDG2: “End hunger, achieve food security, improve nutrition, and promote sustainable agriculture”.

## 9. Applications in the Production of Biochemicals and Pharmaceutics

Immunoglobulins are produced by B-cells and play a key role in the immune system, constituting humoral immunity. These molecules can bind to unknown external molecules because their genes are arranged appropriately in B-cells to produce immunoglobulins, which are specific to different antigens. Immunoglobulins have been widely utilized in biochemistry and biotechnology. For example, immunoglobulins are used in western blotting analysis to detect antigenic protein transferred on a membrane after SDS-polyacrylamide gel electrophoresis [112]. Flow cytometry analysis also uses immunoglobulins to investigate and sort cells expressing antigens against the immunoglobulins [113]. Moreover, antibody drugs based on immunoglobulin molecules have been developed and widely used in clinical settings. In fact, the use of antibody drugs in the treatment of rheumatoid arthritis and several types of cancer has significantly increased in recent years [114,115,116]. Thus, there is a rising need for immunoglobulins in biochemical research and pharmaceutical production. Novel methods for capturing immunoglobulin molecules and producing immunoglobulin-fused proteins have been developed using molecular display technology, as detailed below.

**Figure 6:**
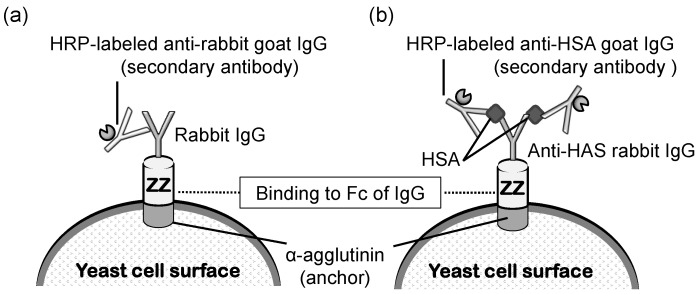
Schematic illustration of (**a**) ELISA to detect rabbit IgG and (**b**) sandwich ELISA to detect HSA using yeast cells displaying ZZ. The first antibody (rabbit IgG) binds to ZZ using its Fc region. *HRP Horseradish peroxidase*, *IgG immunoglobulin G*, *HSA human serum albumin*. The figure was adapted from [119].

Protein A was the first protein to be displayed on the yeast cell surface for capturing immunoglobulin molecules [117]. In general, protein A has been utilized as an attached form to a carrier or beads to purify immunoglobulin from serum samples. A mixture solution derived from blood samples is generally applied on protein A-conjugated Sepharose and flowed in a column [118]. Protein A can capture immunoglobulins by binding to the Fc portion of an immunoglobulin. Next, the column is washed, and purified immunoglobulin is eluted using an appropriate mobile phase liquid. In addition to this achievement with protein A, it has been shown that yeast cells can be converted into immunobeads by displaying protein A on the cell surface [119]. A yeast strain displaying protein A was proven to be useful in an enzyme-linked immunosorbent assay (ELISA), wherein immunoglobulin needs to be fixed to react with the antigen in the solution (Figure 6). Additionally, since protein A on yeast was shown to bind to antibodies from a wide range of species, it was demonstrated that protein A displayed on yeast cells can be applied in sandwich ELISA detection (Figure 6b).

The industrial production of immunoglobulins is most commonly conducted via animal immunization; thus far, animal immunization has been performed using antigen molecules. Generally, animal maintenance and the purification process are very costly. If in vitro production of immunoglobulins or microbial production becomes possible, the production cost and time will be significantly reduced. To attain microbial production and purification of IgG using protein A, the ZZ-domain of protein A was displayed on the yeast surface, and its effects were examined. The Z-domain is derived from the B-domain of protein A and fused tandemly as the ZZ-domain. In this investigation, the fusion protein EGFP-Fc was produced by another yeast strain and co-cultivated with the yeast displaying the ZZ-domain [46,120]. The results demonstrated that the EGFP-Fc secreted into the medium was effectively recovered by the surface-displayed ZZ-domain during cultivation. This novel synergistic production of the Fc fusion protein can provide a convenient and lower-cost production system for secreted recombinant proteins, including immunoglobulins, by microbial cells in biotechnological and pharmaceutical industries. Since these findings will be beneficial in improving the economic activities of the pharmaceutical industry, protein A-displaying yeast can contribute to SDG3: “healthy lives and promotion of well-being for all at all ages”.

## 10. Application in Oral Vaccine Development

The main cause of human death worldwide is infectious diseases. Since the recent COVID-19 pandemic, the majority of human populations have been paying even more attention to and are more conscious of infectious diseases [121]. However, humans have been struggling not only to combat severe acute respiratory syndrome coronavirus 2 of COVID-19 but also many other types of pathogenic microorganisms, including the three major infectious diseases: malaria, tuberculosis, and acquired immunodeficiency syndrome (AIDS) [122,123]. Unfortunately, after one pandemic ends, human populations have to fight another destructive pathogen. The current era of globalization has accelerated the mass transportation and transition of individuals. As a result, the threat of infectious diseases has increased. The development of a convenient tool for preventing various infectious diseases would lead us to achieve SDG3.

Tools for developing oral vaccines have been developed using yeast molecular display, as introduced below. Candidiasis was chosen as a model disease for oral vaccine development. Candidiasis is caused by the fungus *Candida* spp. [124], and its antigen was displayed on the cell surface of *S. cerevisiae*. Systemic or superficial candidiasis is observed when the host immunity is impaired by AIDS or by the administration of immunosuppressants for organ transplantation or cancer chemotherapies [125]. The treatment of candidiasis usually involves the administration of antifungal drugs. Unfortunately, these drugs often cause adverse side effects and generate mutants with reduced susceptibility to antifungal drugs. Furthermore, diagnosis of candidiasis in the earlier stages of infection is difficult; therefore, the disease may have seriously progressed by the time it is diagnosed. Considering these situations of limited clinical options, the prevention of candidiasis by vaccine is thought to be an effective strategy.

To develop an oral vaccine against candidiasis, an antigenic protein of *Candida albicans* was selected to be displayed in yeast [126]. Eno1p was selected as an antigen against *C. albicans* because it well known to be effective in inducing immunological responses [127]. The plasmid pULD1-eno1 contains the fusion gene encoding Eno1p, and 3′-half of α-agglutinin was constructed using an α-agglutinin-based display system in the same way as that in other applications. Yeast displaying Eno1p on its surface was produced by introducing the above plasmid into the BY4741 strain. The resulting strain was named Eno1-SC, whereas the strain with the BY4741-harboring control plasmid, i.e., vacant pULD1, was named Ctrl-SC. Both strains were administered to mice via the oral route.

**Figure 7:**
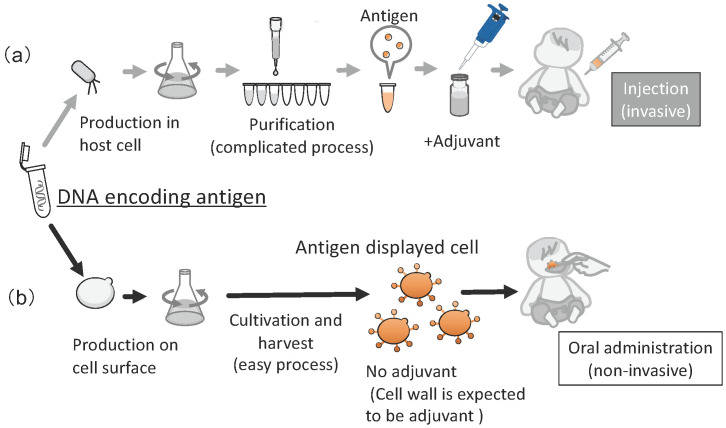
Overview of production of a vaccine by conventional method and molecular display. (**a**) Conventional recombinant vaccine (invasive administration), (**b**) Oral vaccine produced by molecular display using yeast cell (noninvasive administration). The figure was adapted from [126,129].

The average titer of antibody against Eno1p after oral administration of yeast cells displaying Eno1p (Eno1-SC) was 5.2 × 10^3^. Next, a survival test was performed on the orally immunized mice by administering an injection of a lethal dose of *C. albicans*. At 4 weeks after this injection, 60% of the mice that received Eno1-SC survived, whereas all mice that received Ctrl-SC died. Additionally, only 12.5% and 25% of the recombinant Eno1p-vaccinated mice via the subcutaneous and intranasal routes survived at 4 weeks after injection with a lethal dose of *C. albicans,* respectively. In addition, *Lacticaseibacillus casei* (*Lactobacillus casei*) was investigated as a host for cell-surface display of Eno1p. However, the vaccine efficacy was lower than when yeast was used [128,129]. These studies suggested that molecular display of immunogens on yeast cells can provide convenient methods for production of oral vaccines against other infectious diseases. If a gene encoding an effective antigenic protein is known, this system can be used to generate an oral vaccine against its pathogen with no purification steps (Figure 7). With continuous development, this molecular display technology can contribute to promoting human health worldwide, i.e., SDG3, since vaccine production would then not require expensive facilities.

Animal vaccines have also been gaining importance. Recently, fish vaccines have seen increasing use in aquaculture, raising the need for convenient administration methods. Infectious hematopoietic necrosis virus (IHNV) infects farmed rainbow trout (*Oncorhynchus mykiss*) and various salmonid fish species [130]. At present, IHNV is considered to be a serious threat in the salmon industry worldwide since infectious hematopoietic necrosis is a deadly viral disease with a high mortality rate. Currently, there is no decisive solution to this problem. IHNV induces the death of lymphocytes in infected fish. However, it has been shown that this virus binds to a cell receptor through the G protein in its envelope, and an anti-G protein antibody has the ability to neutralize IHNV [131]. Therefore, G protein was displayed on the yeast cell surface [132], and cultured yeast cells were orally administered to a fry of trout. After immunization, a challenge test using IHNV was performed, and the results showed that half of the fish in the immunized group were alive, whereas all fish in the control group (administered yeast without G protein displayed) died 2 weeks after the start of the challenge test. Molecular display of antigens of other fish pathogens has also been developed [133]. Further trials are needed to formulate a robust fish vaccine using molecular display technology, which will contribute to the achievement of SDG14: “Conservation and sustainable use of the oceans seas and marine resources”.

## 11. Summary

In this review, we summarize the relationship between the application of yeast molecular display technology and the concurrent achievement of SDGs. This biotechnology is expected to help achieve a sustainable society. Although this technology currently does not cover all SDGs, its ability and application can be enhanced to realize holistic sustainable development in various fields.

Although this review outlines molecular display technologies only for *S. cerevisiae*, other yeasts such as *Komagataella pastoris* (*Pichia pastoris*) and *Yarrow lipolitica* have also been developed as possible hosts for recombinant protein production [134,135]. For example, the lipase Lip2 was displayed on the surface of *Y. lipolitica* [136], and the investigation showed that Lip2 exhibited higher stability in organic acids and detergents, such as EDTA, SDS, dimethyl sulfoxide, and Tween 80. These properties are important for the application of recombinant proteins in bioprocesses.

Each yeast species and strain has different advantages in molecular display. Thus, the selection of the optimal yeast hosts and genes to be displayed is important for creating a powerful tool that can help us achieve sustainable development. However, almost all the technologies introduced here have been laboratory scale investigations using flasks or microtubules. Therefore, a plant scale investigation and an examination on energy consumption for each application of molecular display technology should also be performed to further progress towards a sustainable society.

## Data Availability

Not applicable.
